# Daily Optogenetic Stimulation of the Left Infralimbic Cortex Reverses Extinction Impairments in Male Rats Exposed to Single Prolonged Stress

**DOI:** 10.3389/fnbeh.2021.780326

**Published:** 2021-12-20

**Authors:** Lucas Canto-de-Souza, Peyton G. Demetrovich, Samantha Plas, Rimenez R. Souza, Joseph Epperson, Krista L. Wahlstrom, Ricardo Luiz Nunes-de-Souza, Ryan T. LaLumiere, Cleopatra Silva Planeta, Christa K. McIntyre

**Affiliations:** ^1^Laboratory of Pharmacology, School of Pharmaceutical Sciences, São Paulo State University – UNESP, Araraquara, Brazil; ^2^Institute of Neuroscience and Behavior, Ribeirão Preto, Brazil; ^3^School of Behavior and Brain Sciences, The University of Texas at Dallas, Richardson, TX, United States; ^4^Texas Biomedical Device Center, The University of Texas at Dallas, Richardson, TX, United States; ^5^Department of Psychological and Brain Sciences, University of Iowa, Iowa City, IA, United States; ^6^Joint Graduate Program in Physiological Sciences, Universidade Federal de São Carlos - UFSCar/UNESP, São Carlos, Brazil; ^7^Iowa Neuroscience Institute, University of Iowa, Iowa City, IA, United States

**Keywords:** memory, PTSD, posttraumatic stress disorder, anxiety, lateralization and brain functions

## Abstract

Post-traumatic stress disorder (PTSD) is associated with decreased activity in the prefrontal cortex. PTSD-like pathophysiology and behaviors have been observed in rodents exposed to a single prolonged stress (SPS) procedure. When animals are left alone for 7 days after SPS treatment, they show increased anxiety-like behavior and impaired extinction of conditioned fear, and reduced activity in the prefrontal cortex. Here, we tested the hypothesis that daily optogenetic stimulation of the infralimbic region (IL) of the medial prefrontal cortex (mPFC) during the 7 days after SPS would reverse SPS effects on anxiety and fear extinction. Male Sprague-Dawley rats underwent SPS and then received daily optogenetic stimulation (20 Hz, 2 s trains, every 10 s for 15 min/day) of glutamatergic neurons of the left or right IL for seven days. After this incubation period, rats were tested in the elevated plus-maze (EPM). Twenty-four hours after the EPM test, rats underwent auditory fear conditioning (AFC), extinction training and a retention test. SPS increased anxiety-like behavior in the EPM task and produced a profound impairment in extinction of AFC. Optogenetic stimulation of the left IL, but not right, during the 7-day incubation period reversed the extinction impairment. Optogenetic stimulation did not reverse the increased anxiety-like behavior, suggesting that the extinction effects are not due to a treatment-induced reduction in anxiety. Results indicate that increased activity of the left IL after traumatic experiences can prevent development of extinction impairments. These findings suggest that non-invasive brain stimulation may be a useful tool for preventing maladaptive responses to trauma.

## Introduction

The medial prefrontal cortex (mPFC) maintains vast connections with both cortical and subcortical structures (McKlveen et al., 2015; Porter and Sepulveda-Orengo, 2020) and contributes to the regulation of emotions (Kesner and Churchwell, 2011) and stress-related responses (Cerqueira et al., 2008). Functional imaging studies in humans indicate that the mPFC is recruited during fear extinction (Phelps et al., 2004) and that patients with post-traumatic stress disorder (PTSD) have reduced mPFC activity during trauma recall (Bremner et al., 1999; Gold et al., 2011), and impaired recall of fear extinction (Milad et al., 2008, 2009). Lesions or inhibition of the mPFC impair extinction recall in fear conditioned rodents (Quirk et al., 2000; Santini, 2004; Kim et al., 2016), whereas stimulation facilitates extinction learning (Milad et al., 2004; Do-Monte et al., 2015), suggesting that mPFC plays a critical role in fear extinction.

Memories from arousing experiences have an important adaptive value, changing the individual’s defensive repertoire and increasing the chances of survival when facing danger in future experiences. However, following a traumatic event, a subset of individuals suffers from persistent pathological symptoms such as those seen in PTSD (Kessler, 1995). Previous trauma is a factor that increases risk of falling into that subset (Breslau et al., 1999). The single prolonged stress (SPS) model for PTSD appears to mimic in rodents the stress that would predispose individuals to impaired extinction following a later trauma (Liberzon and Young, 1997; Knox et al., 2012a; Lisieski et al., 2018). The SPS procedure involves a series of successive stressors in a single day followed by a 7-day quiescent period, and it leads to many PTSD-like behavioral and physiological symptoms (Liberzon and Young, 1997; Kohda et al., 2007; Milad et al., 2009; Glover et al., 2011; Norrholm et al., 2011; Knox et al., 2012a,^2016^; Noble et al., 2017). Behavioral and neuroendocrine alterations following SPS in rodents involve abnormal functioning in brain structures such as the mPFC (Knox et al., 2010; Lim et al., 2017; Souza et al., 2017). Indeed, after a 7-day incubation period in which animals are left undisturbed following SPS exposure, rats show increased anxiety-like behavior and impaired extinction learning, along with reduced glutamate levels in the infralimbic (IL) portion of the mPFC (Knox et al., 2010, 2016; Lim et al., 2017; Piggott et al., 2019; Nawreen et al., 2021).

Together, these findings indicate that IL hypofunctioning during the 7-day incubation period following SPS is associated with subsequent behavioral impairments, suggesting that increasing IL activity following SPS could prevent the impairments in anxiety and extinction. Moreover, previous findings indicate that stress can produce asymmetrical changes in the mPFC, and functional lateralization of mPFC affects emotional states following stress (Sullivan and Gratton, 1999; Cerqueira et al., 2008; Lee et al., 2015; Costa et al., 2016; Victoriano et al., 2020; Santos-Costa et al., 2021) and hemispheric asymmetry in volume of the hippocampus, amygdala, and cortex of PTSD patients has been reported (Adami et al., 2006; Karl et al., 2006; Morey et al., 2012; Zach et al., 2016; Cwik et al., 2020), suggesting that these SPS-induced changes may be lateralized. Previous studies have found that bilateral optogenetic stimulation of the IL or IL inputs to the basolateral amygdala during extinction training enhance extinction memories in rodents (Bukalo et al., 2015, 2021; Do-Monte et al., 2015). These studies have provided insights into the neural substrates of extinction learning and memory using animal models of conditioned fear and exposure. The present work advances understanding of trauma effects on the IL by using daily optogenetic stimulation of either the left or the right IL during the 7-day quiescent period between SPS exposure and fear conditioning to determine whether reversing hypoactivity during this period would prevent SPS effects on anxiety-like behavior and fear extinction.

## Materials and Methods

### Animals

Male Sprague-Dawley rats (*n* = 120) (Taconic Laboratories) weighing 275–315 g at the time of surgery were individually housed with food and water *ad libitum* on a 12 h/12 h light-dark cycle (6:00 am lights on). Only male rats were used because previous studies found that SPS did not impair extinction or increase anxiety-like behavior in female rats (Keller et al., 2015; Mancini et al., 2021). Rats were habituated to the vivarium for at least 3 days between arrival and the beginning of procedures. All procedures were approved by the Institutional Animal Care and Use Committee of the University of Texas at Dallas in compliance with the National Institutes of Health guidelines for the care and use of laboratory animals.

### Surgeries

Under isoflurane anesthesia (2–5%), rats were placed into a stereotaxic frame. A syringe (Syringe, Neuros, 7002, 2 μL) was lowered into the left- or the right- IL region of the prefrontal cortex according to stereotaxic coordinates of a brain atlas [2.6 mm anterior to bregma (AP), ± 0.55 mm lateral to the midline for left and right hemispheres (ML), respectively, and 5.2 mm ventral to the skull surface (DV)] (Paxinos and Watson, 2006). Rats were injected with 0.5 μL of virus using a motorized stereotaxic injector (Stoelting QSI™) and the needle was left in place for 10 min before removal. Following microinjection, optic fibers (0.37 NA; 200 μm core; 10 mm length; Inper) were implanted into the IL (AP: + 2.6 mm to bregma; ML: ± 0.55 mm; DV: -5.0 mm) and fixed in place with acrylic cement over skull screws. The rats received injections of the analgesic ketoprofen (5 mg/kg, i.p.), and antibiotic ceftriaxone (4 mg/kg, i.p.) immediately after surgery. Viruses were infused 4 weeks before SPS to ensure sufficient opsin expression during light stimulation (Do-Monte et al., 2015).

### Viruses

The adeno-associated viruses (AAVs; serotype 5) were AAV5-CaMKII-ChR2(E123A)-EYFP-WPRE and AAV5-CaMKII-EYFP-WPRE and were purchased from the University of North Carolina Vector Core. The opsin used was ChR2(E123A), a mutant form of the cation channel channelrhodopsin-2 permitting high-frequency stimulation up to 200 Hz (Gunaydin et al., 2010; Yizhar et al., 2011; Huff et al., 2013). The CaMKII promoter was used to restrict viral expression to glutamatergic neurons (Liu and Jones, 1996; Goshen et al., 2011).

### Single Prolonged Stress Procedure

The SPS procedure used was based on previous studies (Liberzon and Young, 1997; Souza et al., 2017). First, rats were submitted to restraint stress; they were placed in individual disposable pastry bags (16 in, made by Wilton) with the tip cut off to allow airflow to the nose and the larger end tied tight to restrict movement for 2 h. Immediately after, rats in groups of 2–4 were submitted to the forced swim test for 20 min. After 15 min of recuperation on a clean, dry towel, rats were exposed to diethyl ether in an induction chamber (up to 5 min) to induce a brief loss of consciousness. Following SPS, rats were returned to the vivarium and left undisturbed for 7 days of incubation (except during laser delivery). This quiescent period reduces glutamate levels in the mPFC (Knox et al., 2010; Lim et al., 2017) and drives neuroendocrine and behavioral alterations including impaired extinction of conditioned fear (Liberzon and Young, 1997; Liberzon et al., 1999; Phan et al., 2006). The first laser delivery was performed 30 min after SPS. This stimulation procedure was repeated daily, between the hours of 10 am and 4 pm, for 7 consecutive days.

### Laser Delivery

Rats expressing ChR2 in the left or right IL received unilateral stimulation in the left or right IL (left-IL, right-IL), for 15 min each day, using a blue light laser (Shanghai Laser & Optics Century Co., Ltd.). Control rats expressing only eYFP were unilaterally illuminated in the left or right IL for 15 min per day for 7 days, to control for any non-specific effects of laser light or virus delivery. Laser light (473 nm blue light laser, 10 mW at 20 Hz, 2 s trains, every 10 s) was controlled by a pulse generator (Master-8, A.M.P.I) and passed through a patch cable (200 μm Core, 0.39 NA, ThorLabs), 1 × 2 splitter (200 μm core, 0.22 NA, FONT Canada), fiber-optic rotary joint (Doric), patch cord (200 μm Core, 0.39 NA, ThorLabs) and fiber implants (0.37 NA; 200 μm core; 10 mm length; Inper) above the IL. To confirm stimulation was at 10 mW, laser output was tested with a compact power and energy meter (ThorLabs). Rats were habituated to the patch cord during handling. The optical stimulation protocol was adapted from Huff et al. (2013) and Do-Monte et al. (2015).

### Elevated Plus-Maze

The EPM test evokes anxiety/fear-related defensive responses that are pharmacologically validated (Pellow et al., 1985; Carobrez and Bertoglio, 2005). Rats subjected to SPS show increased anxiety-like behavior when tested on the EPM (Han et al., 2014; Liu et al., 2016). Here, the apparatus consisted of a plus-shaped maze elevated above the floor with 2 oppositely positioned closed arms (50 cm × 10 cm), 2 oppositely positioned open arms (50 cm × 10 cm), and a center area (10 × 10 cm). In the beginning, rats were placed in the central area of the maze, facing an enclosed arm. Maze exploration was recorded from above using a video camera for 5 min. The number of entries into open arms, entries into closed arms, and the time spent in the open arms were scored. The percentage of open arm entries (number of entries into the open arm/total number of entries in both arms) and the percentage of time spent in the open arms [(time in the open arms/300 s) × 100] were calculated to assess anxiety. The number of closed arm entries (CE) was used as a measure of locomotor activity (Cruz et al., 1994).

### Auditory Fear Conditioning

Rats underwent auditory fear conditioning (AFC) 24 h after the EPM test, as described previously (Bremner et al., 1999; Noble et al., 2017). Fear acquisition, extinction training, and extinction retention tests were performed in a Plexiglas box (20 × 20 × 20 cm, with stainless steel grid floor, Vulintus, Plano, TX) housed in a sound-attenuated chamber. The conditioned stimulus (CS) was a 9 kHz tone (75 dB SPL, 30 s duration) always presented at an inter-trial interval of 90 s. The unconditioned stimulus (US) was a moderate footshock (1 s, 0.8 mA) delivered through the grid floor. *Fear Acquisition:* In the acquisition session, rats were first exposed to two unpaired CS to assess baseline freezing to the tones. For the remainder of the acquisition session, rats underwent seven CS + US pairings, with the US administered at the end of each 30-s CS presentation. The conditioned fear response (CFR) was measured by the percentage of freezing during the 30 s CS presentation. Freezing was defined as a period of complete immobility and rapid respiration. *Fear Extinction:* Rats underwent fear extinction training 24 h after acquisition of AFC. During extinction training, rats received 20 CS presentations alone. *Extinction retention:* Twenty-four hours after the extinction training session, rats were subjected to a retention test, where conditioned fear was measured during 5 CS presentations alone.

### Data Collection and Analysis

Behavior was recorded using digital video cameras. Freezing was hand scored by at least two observers who were blind to the experimental condition. The EPM videos were scored by using software (X-plo-rat 2005, University of São Paulo) (Tejada et al., 2018).

### Histology

After the end of behavioral tests, rats were anesthetized in an induction chamber with isoflurane (5%) and then placed in a face mask where they were deeply anesthetized under isoflurane. Transcardial perfusion procedures were performed with phosphate-buffered saline (PBS, pH 7.4), followed by phosphate-buffered saline with 4% paraformaldehyde (PFA). Next, the brains were placed in vials containing PFA for 1 h followed by 30% sucrose until the day of sectioning (40 μm) with a cryostat. Slices were mounted on frosted microscope slides (Thermo Scientific™ Ultra Frost™) with antifade mountant (ProLong™ Gold). Only the rats with virus and implant tracks in the IL were included in statistical analysis.

### Experimental Design

**FIGURE 1:**
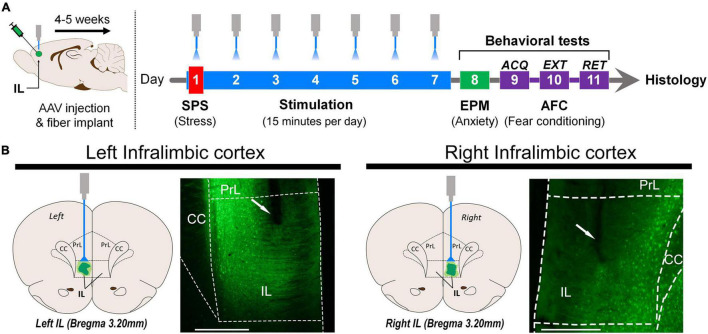
**(A)** Schematic representation of the stereotaxic surgery for AAV virus injection and optical fiber implantation in the left or right infralimbic cortex (IL). Four to five weeks after surgical infusion of the virus, the single-prolonged stress (SPS) procedure was administered in 1 day. Optogenetic stimulation started 30 min after exposure to diethyl ether, and it was given for 15 min every day for 7 days. On day 8, elevated plus maze (EPM) behavior was tested for 5 min. Auditory fear conditioning (AFC): Acquisition (day 9) was followed by one extinction session (day 10). Retention was tested 24 h later (day 11). **(B)** Schematic representation of optical fiber implantation and representative photomicrograph of the left and right infralimbic portion of the medial prefrontal cortex (scale bar = 500 μm). The area below the end of the optical fiber tract was exposed to light. ACQ, acquisition; EXT, extinction; RET, retention; Optical fiber tract (white arrow); PrL, prelimbic cortex; IL, infralimbic cortex; CC, corpus callosum.

Four to five weeks after surgery, rats were subjected to the SPS procedure (Figure 1) and received optogenetic stimulation for 15 min/day for 7 consecutive days during the SPS incubation period. On day 8, the rats were tested for anxiety in the EPM. On day 9, rats underwent acquisition of AFC and, 24 h later, the extinction protocol was performed. Twenty-four hours after extinction training, all rats were tested for extinction retention.

### Statistical Analysis

Data from the left-IL experiment were analyzed using a two-way analysis of variance (ANOVA) or three-way ANOVA (Repeated Measures) [Factor 1—Condition: non-SPS (SPS-) or SPS (SPS+); Factor 2—Optical stimulation: eYFP or ChR2; and Factor 3—Conditioned stimulus: Pre-US and/or trial blocks]. Data from the right-IL experiment were submitted to a Student’s *t*-test or a two-way ANOVA (Repeated Measures) (Factor 1—Optical stimulation: eYFP or ChR2; Factor 2—Conditioned stimulus: Pre-US and/or trial blocks). Significant results were further analyzed using *post hoc* Duncan’s multiple comparisons test. Values of *p* ≤ 0.05 were regarded as significant. Rats with no virus expression or correct optical fiber placement were excluded from the final data analysis.

## Results

Results were analyzed only for the animals in which the tip of the optical fiber was co-localized with viral expression within the IL, as represented by Figure 1B.

### Optogenetic Stimulation of the Left-Infralimbic Region During Single Prolonged Stress Incubation Did Not Alter Anxiety-Like Behavior

**FIGURE 2:**
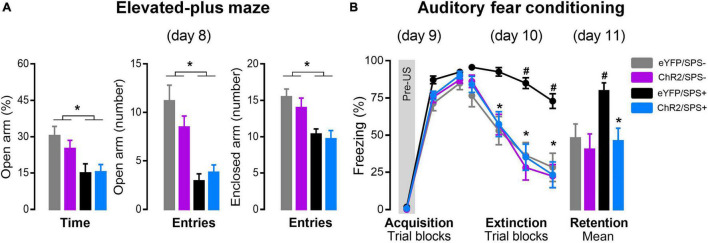
The effects of left-IL optogenetic stimulation on anxiety and extinction of conditioned fear in SPS rats. **(A)** Effect of the SPS on anxiety-like behavior evaluated in the elevated plus-maze (day 8). Bars represent mean + SEM of the percentage of open arm time and the number of open and enclosed arm entries of eYFP/SPS− (*n* = 10), ChR2/SPS- (*n* = 10), eYFP/SPS+ (*n* = 14) and ChR2/SPS+ (*n* = 11) rats. *p < 0.05 vs. SPS- rats. **(B)** Optogenetic stimulation of left IL neurons enhance fear extinction of SPS+ rats. Acquisition (day 9): data are shown as mean ± SEM of the percentage of freezing in blocks of three trials (Pre-US is an average of the two pre-tones and the tone before the shock). Extinction: data are shown as mean ± SEM in blocks of five trials (Total of trials: 20 CS+). Retention: data are shown as mean + SEM of the average of five trials (5 CS+). Duncan’s *post hoc*: *p < 0.05 vs. first trial block (extinction) of each group or eYFP/SPS+ group (retention), and ^#^*p* < 0.05 vs. other groups. Pre-US: baseline (2 CS + no US); SPS-: no single prolonged stress; SPS+: single prolonged stress.

Figure 2A represents the percentage of open arm activity in the EPM of SPS (SPS+) and non-SPS (SPS-) rats after left-IL optogenetic stimulation. A two-way ANOVA indicated significant changes in the exploration of the open arms after SPS, independent of optogenetic stimulation and without factor interaction [Figure 2A% of Open arm: ANOVA, Condition Time: *F*1_(1, 41)_ = 12.017, *p* = 0.001; Optical stimulation Time: *F*2_(1, 41)_ = 0.04, *p* = 0.51; factor interaction Time: *F*1 × *F*2_(1, 41)_ = 0.62, *p* = 0.43; Open arm entries, Condition Entries: *F*1_(1, 41)_ = 37.32, *p* = 0.001; Optical stimulation Entries: *F*2_(1, 41)_ = 0.73, *p* = 0.40; factor interaction Entries: *F*1 × *F*2_(1, 41)_ = 2.87, *p* = 0.10]. A *post hoc* test revealed that SPS+ rats displayed reduced exploratory activity in the open arms compared to SPS- rats. Two-way ANOVA also indicated significant changes in closed arm entries [CE: *F*1_(1, 41)_ = 20.56, *p* = 0.00005], independent of optogenetic stimulation [*F*2_(1, 41)_ = 1.05, *p* = 0.31], and no interaction between factors was detected [*F*1 × *F*2_(1, 41)_ = 0.18, *p* = 0.68]. Although Duncan’s multiple comparisons test showed that SPS+ rats had a significant reduction in enclosed arm entries compared to SPS- rats, potentially indicating an impairment of locomotor activity, a *post hoc* analysis revealed that enclosed arm entries did not covary with percentage of open arm entries [*F*1_(1, 41)_ = 8.96, *p* = 0.005] and time [*F*1_(1, 41)_ = 8.61, *p* = 0.01]. These findings suggest that the number of enclosed arm entries was not an influential factor in the anxiogenic-like behavior induced by the SPS. These results are consistent with previous evidence that SPS increases anxiety-like behavior in rats (Han et al., 2014; Liu et al., 2016; Deslauriers et al., 2018) and suggest that stimulation of the left-IL did not reverse the anxiogenic state induced by SPS.

### Stimulation of Left-Infralimbic Region Neurons Reversed the Impairment in Fear Extinction in Single Prolonged Stress Rats

On the day after the EPM (day 9), rats underwent fear acquisition using AFC (Figure 2B). The results indicate that optogenetic stimulation during the incubation period did not interfere with fear acquisition [Figure 2B Acquisition: ANOVA, stress *F*1_(1, 50)_ = 6.60, *p* = 0.01; conditioned stimulus *F*3_(2, 100)_ = 1638.28, *p* < 0.00001; optogenetic stimulation *F*2_(1, 50)_ = 0.37, *p* = 0.55; factor interaction *F*1 × *F*2 × *F*3_(2, 100)_ = 2.35, *p* = 0.10]. Twenty-four hours later (day 10), rats were subjected to extinction training by receiving 20 presentations of the CS without presentation of the US. A three-way repeated-measures ANOVA indicated significant differences between the three factors (Figure 2B Extinction: ANOVA *F*1_(1, 50)_ = 10.61, *p* = 0.002; *F*2_(1, 50)_ = 9.09, *p* = 0.004; *F*3_(3, 150)_ = 125.90, *p* < 0.00001] but no interactions [*F*1 × *F*2 × *F*3_(3, 150)_ = 1.78, *p* = 0.15]. A *post hoc* test revealed that control stimulation rats that underwent SPS (eYFP/SPS+) displayed significantly higher freezing to the CS during the last two trial blocks compared to control stimulation rats that did not receive SPS (eYFP/SPS-) (*p* ≤ 0.03), indicating an extinction impairment. Conversely, rats that underwent SPS and received stimulation of the left-IL (ChR2/SPS+) showed a significant reduction in freezing in the last two trial blocks compared to controls that underwent SPS (eYFP/SPS+) (*p* ≤ 0.02) suggesting that left-IL stimulation reversed the extinction impairment driven by SPS. Optogenetic stimulation of the left-IL had no effect on extinction learning in non-SPS rats (ChR2/SPS- group).

Twenty-four hours after extinction training, rats were tested in the same context for extinction retention (day 11). A two-way ANOVA indicated significant differences between the condition and optical stimulation factors, and a trend toward an interaction between them [Figure 2B Retention: ANOVA, *F*1_(1, 50)_ = 4.33, *p* = 0.04; *F*2_(1, 50)_ = 7.94, *p* = 0.007; *F*1 × *F*2_(1, 50)_ = 3.58, *p* = 0.06]. A *post hoc* test revealed no difference between eYFP/SPS- and ChR2/SPS+ groups (*p* = 0.60), and that eYFP/SPS+ rats displayed significantly higher freezing to the CS during the extinction retention compared to the other groups (*p* ≤ 0.007).

### Stimulation of the Right-Infralimbic Region Neurons Did Not Alter Anxiety-Like Behavior and Had No Effect on Fear Extinction of SPS+ Rats

**FIGURE 3:**
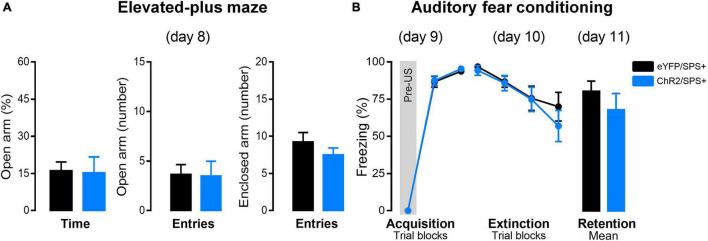
The effects of right-IL optogenetic stimulation on anxiety and extinction of conditioned fear in SPS rats. **(A)** Effect of the right-IL optical stimulation of SPS rats on anxiety-like behavior evaluated in the elevated plus-maze (day 8). Bars represent mean + SEM of the percentage of open arm time and the number of open and enclosed arm entries of eYFP/SPS+ (*n* = 9) and ChR2/SPS+ (*n* = 8) rats. **(B)** ChR2 activation of right IL neurons does not affect fear acquisition, extinction, or retention of SPS+ rats. Acquisition (day 9): data are shown as mean ± SEM of the percentage of freezing in blocks of three trials (Pre-US is an average of the two pre-tones and the tone before the shock. Extinction: data are shown as mean ± SEM in blocks of five trials (Total of trials: 20 CS+). Retention: data are shown as mean + SEM of the average of five trials (5 CS+). SPS-: no single prolonged stress; SPS+: single prolonged stress.

Figure 3A represents the percentage of open arm entries and time as well as closed arm entries in the EPM of rats subjected to SPS and optogenetic stimulation. Student’s *t*-test indicated no differences between both groups [Time: *t*(15) = 0.23, *p* = 0.82; Entries: *t*(15) = 0.10, *p* = 0.93; CE: *t*(15) = 1.07, *p* = 0.30]. These results suggest that optogenetic stimulation parameters applied in the right-IL during the incubation did not alter the anxiogenic state induced by SPS.

Twenty-four hours after the EPM, rats underwent AFC for fear acquisition (day 9) (Figure 3B). A two-way ANOVA indicated that rats from both groups acquired fear conditioning as indicated by a significant increase in freezing during acquisition [*F*2_(2, 30)_ = 1188.42, *p* < 0.00001; *F*1 × *F*2_(2, 30)_ = 0.07, *p* = 0.93]. This data indicates that ChR2 activation of the right infralimbic prefrontal cortex during the incubation period of the SPS does not interfere with the fear acquisition.

On the day after the AFC training (day 10), rats were exposed to extinction training by receiving 20 presentations of the CS. A two-way repeated-measures ANOVA indicated significant differences in freezing between the trial blocks [*F*2_(3, 45)_ = 15.83, *p* < 0.00001]. Conversely, no differences in freezing between groups [*F*1_(1, 15)_ = 0.30, *p* = 0.59] or factor interactions [*F*1 × *F*2_(3, 45)_ = 0.70, *p* = 0.56] were detected.

Twenty-four hours after extinction training, rats were tested in the same context for extinction retention. Student’s *t*-test indicated no differences between both groups [*t*(15) = 0.99, *p* = 0.34]. These results suggest that right-IL optical stimulation during the incubation does not enhance fear extinction in SPS+ rats. Because there was no effect of right-IL stimulation in SPS+ rats, we did not examine effects of right-IL stimulation in SPS- rats.

## Discussion

Enhanced anxiety-like behavior and impaired extinction memory are symptoms observed in male rodents subjected to the SPS procedure (Liberzon and Young, 1997; Kohda et al., 2007; Knox et al., 2012a,^2016^; Noble et al., 2017; Ferland-Beckham et al., 2021). Consistent with previous results, we found that SPS exposure increased anxiety-like behavior and severely impaired extinction of conditioned fear in male rats. The present results also indicate that optogenetic stimulation of the left IL region of the mPFC during the incubation period of the SPS protocol reversed SPS-induced extinction deficits, though it did not alter anxiety-like behavior. In contrast, optogenetic stimulation of the right IL had no effect on either measure. These results suggest that left-IL stimulation restores normal mPFC functioning with regard to the extinction of fear-related behavior. It is worth noting that the amygdala is another brain area which has hemispheric asymmetries in processing emotion, fear, stress, and pain in rodents (Baker and Kim, 2004; Adamec et al., 2005; LaLumiere, 2005; Allen et al., 2021).

Prior work indicates that the SPS protocol induces behavioral and physiological phenomena similar to those observed in PTSD patients (Liberzon and Young, 1997; Blechert et al., 2007; Kohda et al., 2007; Glover et al., 2011; Norrholm et al., 2011; Knox et al., 2016). Although the SPS protocol does not impair extinction or increase anxiety-like behavior in female rats (Keller et al., 2015; Mancini et al., 2021), this procedure has been widely used as an animal model of PTSD (Liberzon and Young, 1997; Knox et al., 2016; Souza et al., 2017; Lisieski et al., 2018; Serova et al., 2019; Su et al., 2019; Ferland-Beckham et al., 2021). In addition to increased anxiety and impaired extinction memory consolidation, rats show increased negative hypothalamic–pituitary–adrenal (HPA) feedback (Knox et al., 2012a,^2016^; Han et al., 2014; Liu et al., 2016; Noble et al., 2017; Deslauriers et al., 2018) and altered glucocorticoid receptor expression 7 days after SPS exposure (Liberzon et al., 1999; Wang et al., 2009; Knox et al., 2012b; George et al., 2013). In the present study we found that SPS+ rats showed anxiety-like behavior, characterized by a lower exploration of the open arms of the EPM, when compared to SPS- rats. Although evidence indicates that an undisturbed post-stress incubation period of 7 days prior to behavioral testing is necessary to observe some behavioral and neuronal alterations (Liberzon and Young, 1997; Liberzon et al., 1999; Phan et al., 2006; Knox et al., 2010; Lim et al., 2017), our results suggest that the disturbance associated with sham laser delivery (eYFP group) during the incubation period did not interfere with SPS-induced anxiety-like behavior or extinction impairments.

Previous SPS studies did not show differences in the acquisition of conditioned fear or extinction, but they showed evidence of impaired retention of extinction (Knox et al., 2012a,^2016^; Vanderheyden et al., 2015; Lin et al., 2016, 2020). In the present studies, freezing remained high (up to 70%) in eYFP/SPS+ rats even after 20 extinction trials, in both experiments with eYFP infused into the left or right IL, indicating that SPS interfered with acquisition of extinction. Even though there was not a significant group difference during fear conditioning, this raises the possibility that stimulation of the IL could have attenuated an exacerbation in fear acquisition, in addition to reversing extinction impairments.

The involvement of the mPFC in fear extinction in humans and rodents is well-established (Quirk et al., 2000; Milad et al., 2004, 2009; Santini, 2004) and bilateral optogenetic stimulation of the IL delivered *during extinction acquisition* enhances fear extinction in rats (Do-Monte et al., 2015). Here we found that optogenetic stimulation of the left IL *during the 7-day incubation period* reversed the SPS-induced extinction impairment. However, optogenetic stimulation of the right IL during the incubation period had no effect on the extinction of conditioned fear in SPS-treated rats. These findings suggest that left-IL optical stimulation enhances the resilience of stressed rats.

Studies indicate that SPS reduces neural activity in the mPFC (Knox et al., 2010; Lim et al., 2017; Piggott et al., 2019) and decreases glutamate levels in the IL (Nawreen et al., 2021), but not in the prelimbic (PrL) cortices (Piggott et al., 2019). Although previous SPS investigations did not distinguish between hemispheres, prior work in both rodents and humans suggests that prolonged stress decreases synaptic communication between the mPFC hemispheres characterized by a lower activity and reduction of the left mPFC volume (Cerqueira et al., 2005; Czéh et al., 2008; Lee et al., 2015). Evidence suggests that mPFC lateralization modulates neuroendocrine and autonomic responses to stress (Sullivan and Gratton, 1999). Bilateral or right, but not left, mPFC lesions, attenuate the increase in corticosterone concentrations induced by exposure to stress (Cerqueira et al., 2008). Moreover, chronic stress results in asymmetric volume and functional changes in mPFC, as such stress induces hyperactivity of the right mPFC and hypoactivity of the left mPFC (Cerqueira et al., 2008; Czéh et al., 2008; Lee et al., 2015). Such alterations may be related to loss of resilience and maladaptive response development (Cerqueira et al., 2008).

Prior work using optogenetic approaches has provided insight into IL activity during fear conditioning extinction. Do-Monte et al. (2015) found that bilateral optical stimulation of IL neurons during extinction training enhanced fear extinction. In addition, they found that inhibition had no effect on the extinction acquisition but led to impaired extinction retrieval. Such findings suggest a critical role for the IL in the consolidation of extinction of conditioned fear. In contrast, the present work provided IL stimulation during the incubation period and not in conjunction with extinction training and such stimulation was delivered unilaterally, rather than bilaterally. This stimulation delivered to the left-IL did not alter freezing as assessed in the first trial block of extinction. However, SPS rats that received left-IL stimulation displayed normal acquisition of extinction, as assessed by the third and fourth blocks, in contrast to the SPS rats that did not receive stimulation. Moreover, the SPS rats receiving stimulation did not differ from rats that did not receive SPS. Stimulation of the left IL before fear conditioning did not enhance extinction in rats that were not exposed to SPS, suggesting that optical stimulation of the left IL before fear conditioning is not sufficient to enhance extinction in normal rats.

Exposure to SPS affected time spent in the open arms of the elevated plus maze, suggesting that the trauma of SPS increased anxiety-like behavior. Several findings suggest that left dorsal mPFC (dmPFC) can exert an inhibitory role over the right dmPFC, attenuating the anxiogenic effects driven by stressors (Cerqueira et al., 2008; Costa et al., 2016; Victoriano et al., 2020; Santos-Costa et al., 2021). However, other evidence suggests that enhanced excitability of the IL decreased time spent in the center of an open field and in open arms of an EPM (Bi et al., 2013) or had no effect (Suzuki et al., 2016). Moreover, bilateral IL continuous light stimulation over 60 min 1 day prior to testing reduced latency to eat in a novelty-suppressed feeding task, indicating an anxiolytic effect (Fuchikami et al., 2015), whereas acute left-IL optogenetic stimulation during behavioral tests enhanced anxiety-like behaviors (Berg et al., 2019) or did not disrupt anxiety-like responses (Covington et al., 2010). The present findings that left-IL optogenetic stimulation does not prevent the SPS-induced decrease in time spent exploring open arms of the EPM may be due to the functional differences between IL and dmPFC in the expression of anxiety-like behaviors (Santos-Costa et al., 2021), differences in unilateral vs. bilateral stimulation, or to differences in the timing of stimulation. Given the lack of a measurable effect of left IL stimulation on anxiety-like behavior in the present study, it is unlikely that the effect of optogenetic stimulation of the left IL on extinction is due to a general reduction in anxiety levels.

The present findings indicate that optogenetic stimulation of the left IL during the 7 days after SPS prevented SPS-induced impairments in extinction of conditioned fear. This is consistent with evidence that SPS reduces mPFC activity and glutamate levels in the IL. However, 15 min of light-driven activation of CaMKII-expressing neurons every day for 7 days is not likely to mimic IL activity in animals who were not exposed to SPS. Based on the promising results of Do-Monte et al. (2015) showing that optogenetic stimulation of the IL during extinction training enhanced extinction memory, we used the same stimulation frequency in SPS-treated rats, but shifted the stimulation to the time period that is implicated in the development of extinction impairments and degeneration of the IL. Whether this unilateral optogenetic stimulation corrected glutamate levels or general IL function remains to be seen. Furthermore, it is unclear whether daily stimulation was necessary, or if a single stimulation after SPS would be sufficient to prevent the effect of SPS on extinction memory. A study by Lobo et al. (2013) demonstrated that optogenetic stimulation of synaptic inputs to the striatum for 10 min/day for 5 days induced expression of the synaptic plasticity marker ΔFosB in specific striatal medium spiny neurons, and Ahmari et al. (2013) demonstrated that daily optical stimulation of orbitofrontal projections to the ventromedial striatum for 5 min/day for 6 days, induced long-term changes in grooming behavior, a mouse behavior that is related to obsessive compulsive disorder. These findings suggest that repeated optogenetic stimulation produces lasting brain changes. Future studies will systematically investigate the timing requirements of optogenetic stimulation of the IL after SPS exposure, and associated changes in IL activity and morphology.

Although SPS does not impair extinction of conditioned fear in female rats (Keller et al., 2015), a recent study showed that SPS exposure does increase anxiety-like behavior on the elevated plus maze and depressive-like behavior on the forced swim test, in female rats (Nahvi et al., 2019), suggesting that SPS studies could be used to better understand sex differences that are seen across species. Consistent with findings from studies of SPS in rats, a laboratory study measuring extinction of conditioned fear in human PTSD patients showed that men exhibited impaired extinction recall but women with PTSD did not (Shvil et al., 2014). The findings of this study further indicated that the PFC was differentially activated during fear extinction recall in men and women with PTSD. Findings of a recent rodent study suggest that estradiol enhances consolidation of extinction memory by shifting the interactions between the IL and centromedial amygdala (Maeng et al., 2017). Given the sex differences in susceptibility to PTSD in humans, and the sexual dimorphism of the brain regions involved, SPS and other rodent models may be used to investigate differences in trauma effects on the brain and behavior. Although the present study did not include female subjects, future studies should probe these sex differences for the purposes of understanding the pathophysiology associated with specific trauma-related symptoms and development of appropriate, potentially sex-specific treatments.

The present results indicate that hemispheric functional lateralization of the IL region of the mPFC might be involved in the capacity of resilience after a traumatic experience. Using daily optogenetic stimulation of the left IL, we prevented the extinction impairments produced by exposure to SPS. These findings suggest that stimulation of the ventromedial prefrontal cortex, such as with transcranial stimulation subsequent to a traumatic event, could serve as a prophylactic to prevent maladaptive fear.

## Data Availability Statement

The raw data supporting the conclusions of this article will be made available by the authors, without undue reservation.

## Ethics Statement

The animal study was reviewed and approved by the University of Texas at Dallas, Institutional Animal Care and Use Committee.

## Author Contributions

LC-D-S, RS, RN-D-S, CP, and CM conceived the idea. LC-D-S, PD, and SP performed the experiments and analyses. JE performed the technical support for optimizing data collection. LC-D-S, PD, SP, RS, KW, RL, and CM wrote the manuscript. CM supervised the study. All authors discussed the results and commented on the manuscript.

## Conflict of Interest

The authors declare that the research was conducted in the absence of any commercial or financial relationships that could be construed as a potential conflict of interest.

## Publisher’s Note

All claims expressed in this article are solely those of the authors and do not necessarily represent those of their affiliated organizations, or those of the publisher, the editors and the reviewers. Any product that may be evaluated in this article, or claim that may be made by its manufacturer, is not guaranteed or endorsed by the publisher.
